# Identification of an eight-m6A RNA methylation regulator prognostic signature of uterine corpus endometrial carcinoma based on bioinformatics analysis

**DOI:** 10.1097/MD.0000000000027689

**Published:** 2021-12-10

**Authors:** Chenyun Miao, Xiaojie Fang, Yun Chen, Ying Zhao, Qingge Guo

**Affiliations:** a Department of TCM Gynecology, Hangzhou TCM Hospital Affiliated to Zhejiang Chinese Medical University, Hangzhou, China; b Department of Anorectal Surgery, Hangzhou TCM Hospital Affiliated to Zhejiang Chinese Medical University, Hangzhou, China; c Department of TCM, Hangzhou Women's Hospital, Hangzhou, China.

**Keywords:** bioinformatics, M6A RNA methylation regulator, prognostic signature, uterine corpus endometrial carcinoma

## Abstract

N6-methyladenosine (m6A) methylation is proved to play a significant role in human cancers. This study aimed to explore the association between m6A ribonucleic acid (RNA) methylation regulators and uterine corpus endometrial carcinoma (UCEC), and build a prognostic signature of m6A regulators for UCEC.

RNA-seq transcriptome data and clinicopathological data of UCEC were downloaded from the Cancer Genome Atlas database. We compared the expression of 23 m6A-regulators in tumor tissues and nontumor tissues. Then we classified the data into 3 clusters by consensus clustering analysis. Several regulators were picked out as the prognostic signature of patients with UCEC based on least absolute shrinkage and selection operator Cox regression analysis. Additionally, we established a predictive nomogram to calculate survival times. Finally, we used receiver operating characteristic curve, univariate Cox regression analysis, and multivariate Cox regression analysis to further verify the prognostic value of the risk signature consisting of m6A regulators.

The expression of 18/23 m6A regulators was significantly different in UCEC compared with normal samples. Gene ontology functional analysis of these regulators revealed that they were mainly participated in RNA splicing, stabilization, modification, and degradation. LRPPRC, IGFBP2, KIAA1429, IGFBP3, FMR1, YTHDF1, METTL14, and YTHDF2 were selected to construct the risk signature and predictive nomogram. The results of receiver operating characteristic curve, univariate Cox regression analysis, and multivariate Cox regression analysis for the risk signature showed a good predictive performance for UCEC.

The risk signature of 8-m6A regulators has potential prognostic value for patients with UCEC.

## 1 Introduction

Uterine corpus endometrial carcinoma (UCEC) is one of the most frequent malignant female reproductive tumors with high incidence and mortality.^[1]^ Recently, the incidence of UCEC has been on a steady rise due to increased aging and the escalating global obesity rate, and thus it has become the fourth most common cancer in women.^[2]^ According to data reported by a recent study, the estimated number of new cases of UCEC in the United States was 65,620 in 2020, while the estimated number of deaths was 12,590.^[3]^ In addition, it has been reported that UCEC causes about 76,000 deaths each year worldwide.^[4]^ To date, there is no elaborate therapy for improving UCEC prognosis, especially hormone-dependent type II patients. Recent studies on molecular mechanisms of UCEC have led to the emergence of molecular targeting as an effective strategy for drug development.^[4,5]^ Therefore, this calls for the identification of new prognostic biomarkers and molecular targets to predict outcome for UCEC patients and guide individualized therapy.

Ribonucleic acid (RNA) modification can influence gene expression programs profoundly. N6-methyladenosine (m6A) modification is the most prevalent form of methylation modification in messenger RNA (mRNA) and noncoding RNA of eukaryotic species, which modulates RNA splicing, translation, and other biological processes.^[6]^ A previous study reported that m6A RNA methylation regulators, including methyltransferase, m6A-binding proteins, and demethylases, regulate tumor proliferation, migration, and invasion.^[7]^ Liu et al^[8]^ revealed that reductions in m6A methylation occur 70% of endometrial cancers. Moreover, Fat mass and obesity-associated gene (*FTO*), a m6A regulator, can catalyze demethylation modification in 3’UTR region of HOXB13 mRNA to promote endometrial tumour metastasis and invasion.^[9]^ These researches all show that m6A has a regulatory effect on endometrial carcinoma.

In this study, we used bioinformatics analysis to investigate the predictive value of m6A regulators on UCEC prognosis. First, we analyzed gene expression profiles of UCEC samples retrieved from the Cancer Genome Atlas (TCGA) database and matched clinical information. We found that the expression levels of 18 m6A regulators were significantly different in UCEC patients. Based on the Lasso model, we established an eight-m6A regulators risk signature, which could effectively predict the prognosis of UCEC patients.

## 2 Materials and methods

### 2.1 Acquisition of data

All RNA-seq transcriptome profiling data and clinical data were searched and downloaded from the TCGA database (https://cancergenome.nih.gov/). In total 552 UCEC tumor samples and 23 normal samples were obtained.

### 2.2 Selection of M6A RNA methylation regulators

According to Shen et al and Deng et al,^[10,11]^ we selected 23 m6A RNA methylation regulators, including methyltransferase like “writer” (*METTL3, METTL14, METTL16, RBM15, RBM15B, WATP, ZC3H13,* and *KIAA1429*), m6A-binding proteins like “reader” (*IGF2BP1, IGF2BP2, IGF2BP3, YTHDF1, YTHDF2, YTHDF3, YTHDC2, YTHDC1, HNRNPC, HNRNPA2B1, FMR1, LRPPRC,* and *RBMX*), and demethylases like “eraser” (*FTO* and *ALKBH5*).

### 2.3 Bioinformatics analysis

First, we merged the RNA-seq transcriptome data and extracted information of the 23 m6A methylation regulators using Perl package (version strawberry-perl-5.30.1.1-64bit). Next, we compared the expression levels of 23 regulators in 552 UCEC tumor samples and 23 normal samples, and then draw heatmaps and violin plots via R (version 4.0.3) software to visualize differential expression. Furthermore, Gene Ontology (GO) analysis was performed for functional annotation of the differentially expressed genes.

To evaluate the association between m6A regulators and the clinical prognosis of UCEC patients, we divided the samples into different groups in accordance with consistent clustering algorithm. Then we used the least absolute shrinkage and selection operator (LASSO) Cox regression analysis to identify m6A regulators associated with patient survival rate to develop a risk signature for UCEC and determine the final risk score. Taking into account the risk score, we classified patients into low-risk group and high-risk group, and compared the overall survival (OS) of these groups. Subsequently, receiver operating characteristic (ROC) curves, univariate, and multivariate Cox regression analyses were performed to verify the prediction accuracy of the risk signature. Next, we constructed the signature as a predictive nomogram to estimate the 1-year survival, 2-year survival, and 3-year survival of the patients.

### 2.4 Statistical analysis

All statistical analyses were performed using R software. Kaplan–Meier method was used to analyze OS, while the chi-square test was used to analyze the correlation between risk signature and clinical characteristics. In addition, univariate and multivariate Cox regression analyses were used to determine the prognostic value of risk signature. Moreover, the prediction accuracy of the prognostic signature was evaluated using ROC and area under the curve (AUC). *P* < .05 was considered a statistically significant difference.

### 2.5 Ethical statements

As all data were obtained from public database, this study did not require ethical approval.

## 3 Results

### 3.1 Expression of M6A RNA methylation regulators in UCEC samples

**Figure 1:**
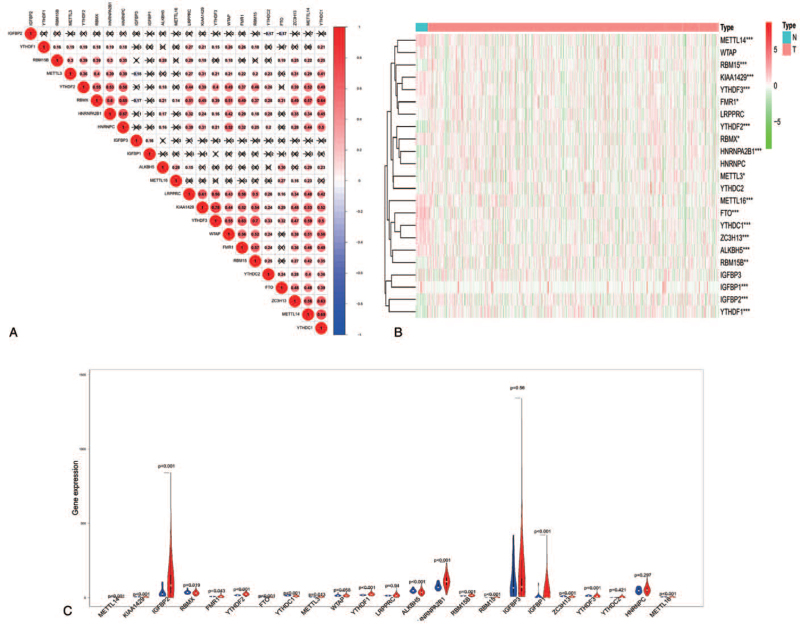
Expression of 23 m6A RNA methylation regulators in UCEC samples. A, Correlation analysis of 23 m6A RNA methylation regulators. The red represents positive correlation; the blue represents a negative correlation. B, The expression heat map of 23 m6A RNA methylation regulators in UCEC samples. ^∗^*P* < .05, ^∗∗^*P* < .01, and ^∗∗∗^*P* < .001. C, The violin plots revealed expression of m6A RNA methylation regulators in UCEC samples. m6A = N6-methyladenosine, RNA = ribonucleic acid, UCEC = uterine corpus endometrial carcinoma.

First, we explored the correlation among the 23 m6A regulators and found that most of these regulators had a direct positive correlation. Among them, the tightest interactions were between *KIAA1429* and *YTHDF3*, explained 0.78 (see Fig. 1A). However, there was no correlation between *IGFBP1* and the other m6A regulators, and negative correlations were observed for *IGFBP2* and *IGFBP3* with other m6A regulators (see Fig. 1A). To further investigate the relation between m6A RNA methylation regulators and UCEC, we analyzed the RNA-seq transcriptome profiling data of UCEC patients obtained from the TCGA dataset. Results showed that 18 m6A regulators (*METTL14, RBM15, KIAA1429, YTHDF2, FMR1, YTHDF2, RBMX, HNRNPA2B1, METTL3, METTL16, FTO, YTHDC1, ZC3H13, ALKBH5, RBM15B, IGF2BP1, IGF2BP2,* and *YTHDF1*) were differentially expressed in UCEC samples (see Fig. 1, B and C).

### 3.2 Functional enrichment analysis of M6A RNA methylation regulators

**Figure 2:**
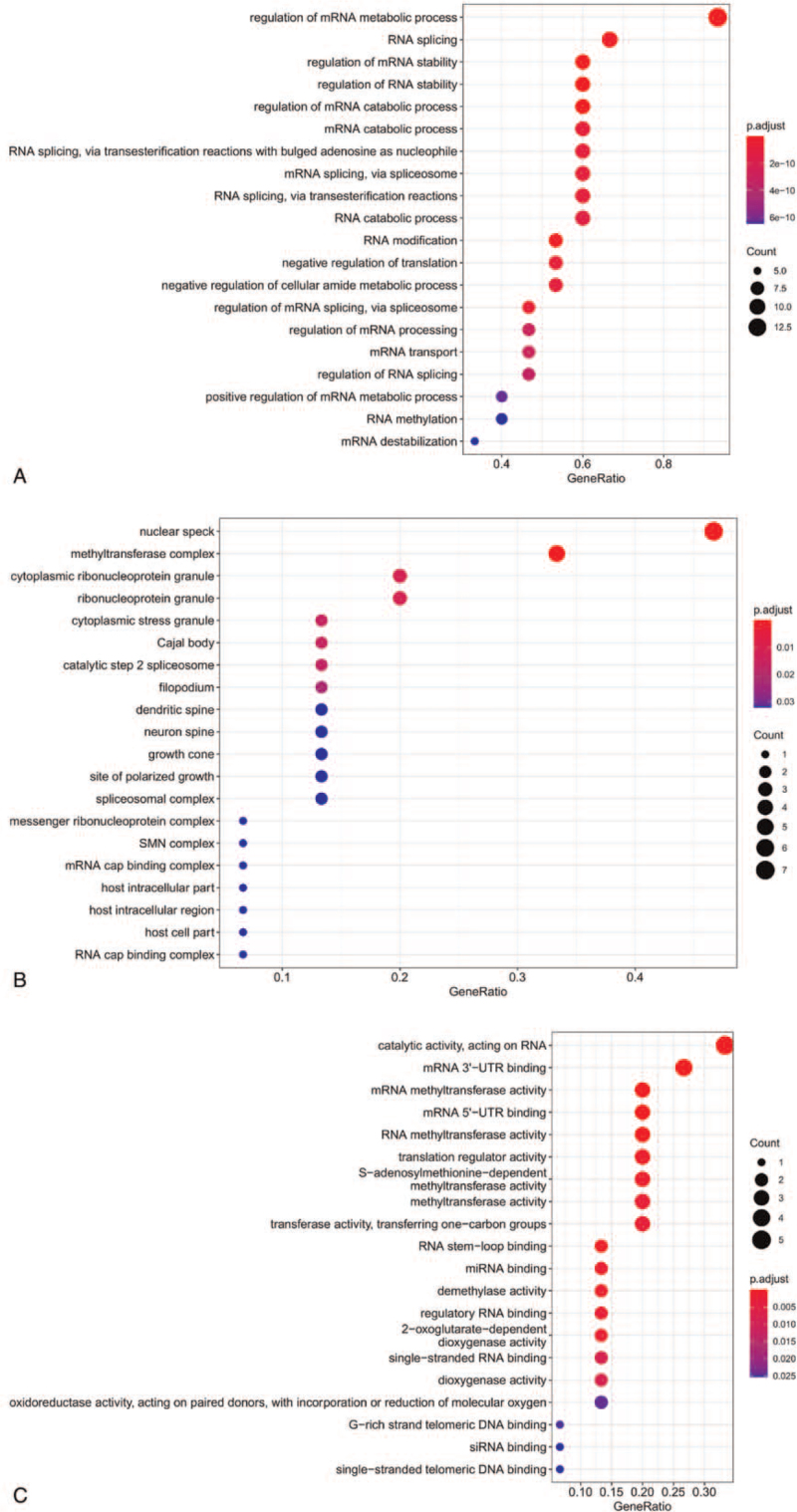
Functional enrichment analysis of differentially expressed m6A regulators. A, BP category of GO analysis of 18 m6A regulators. B, CC category of GO analysis of 18 m6A regulators. C, MF category of GO analysis of 18 m6A regulators. The size of dots is representative of the number of genes. The color represents the *P* value. BP = biological process, CC = cellular components, GO = gene ontology, m6A = N6-methyladenosine, MF = molecular functions.

We used GO analysis to further understand the function of the differentially expressed regulators. With regard to GO analysis, terms in the biological process category focused on RNA modification, splicing, and stability (“regulation of mRNA metabolic process,” “mRNA destabilization,” “RNA destabilization,” “positive regulation of mRNA metabolic process,” “regulation of mRNA stability,” “regulation of RNA stability,” and “regulation of mRNA catabolic process”) (see Fig. 2A). Similarly, terms in the molecular functions category were associated with RNA methylation modification (“mRNA methyltransferase activity,” “mRNA 5’ − UTR binding,” “mRNA 3’−UTR binding,” and “catalytic activity”) (see Fig. 2C). Furthermore, terms in the cellular components category were mainly associated with “nuclear speck,” “methyltransferase complex,” “cytoplasmic ribonucleoprotein granule,” and “ribonucleoprotein granule” (see Fig. 2B).

### 3.3 Three clusters of UCEC determined by consensus clustering of M6A RNA methylation regulators

**Figure 3:**
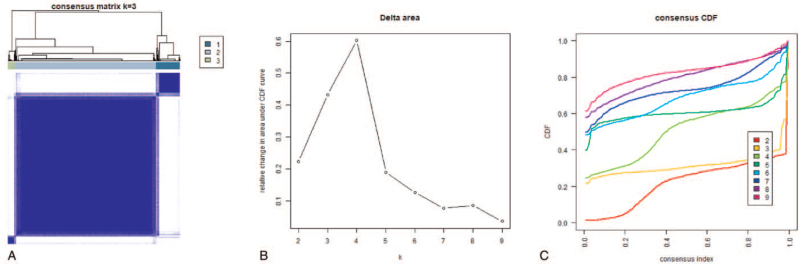
Identification of consensus clusters by m6A RNA methylation regulators. A, Matrix correlation between groups (k = 3). B, Relative change in area under CDF curve for k = 2–9. C, Consensus clustering CDF for k = 2–9. CDF=cumulative distribution function, m6A = N6-methyladenosine, mRNA = messenger RNA.

**Figure 4:**
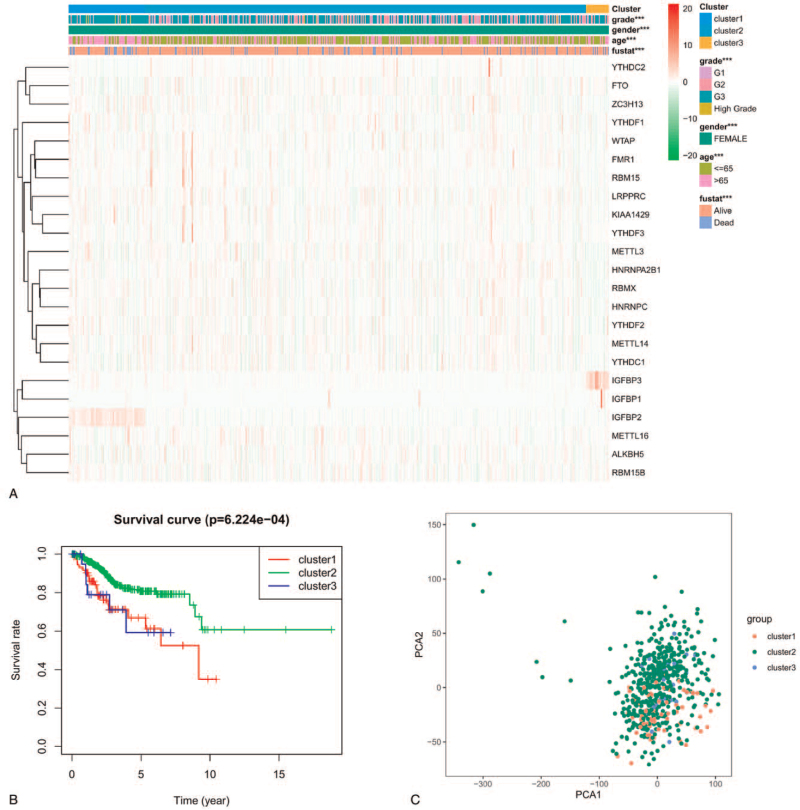
Clinical characteristics and prognosis of UCEC. A, The heat map of 23 m6A regulators expression and clinical characteristics of the 3 clusters. B, Kaplan–Meier overall survival curve of UCEC patients of 3 clusters (cluster 1: blue; cluster 2: blue; clusters 3: orange). C, Principal component analysis of the RNA expression profile in the TCGA dataset (cluster 1: red; cluster 2: green; clusters 3: blue). m6A = N6-methyladenosine, RNA = ribonucleic acid, UCEC = uterine corpus endometrial carcinoma.

We grouped the 552 UCEC samples by consensus clustering. According to Figure 2A and 2B, K=3 was considered the optimal cluster number and thus the samples were divided into 3 groups (see Fig. 3). Comparison of the 23 m6A regulators expression in individual groups was performed. Next, we evaluated other factors obtained from the TCGA dataset, such as age, gender, fustat, and tumor grade. The results revealed that expression levels of the 23 m6A regulators were different in clusters 1, 2, and 3 (see Fig. 4A), and there were significant differences in all clinical factors mentioned above among the 3 groups. As presented in Figure 4B, the OS rate of cluster 2 was longer than that of clusters 1 and 3, indicating better clinical prognosis (*P* = 6.224e−04). Moreover, principal component analysis was executed to confirm that the grouping was feasible (see Fig. 4C).

### 3.4 Establishment of a risk signature consisting of M6A RNA methylation regulators

**Figure 5:**
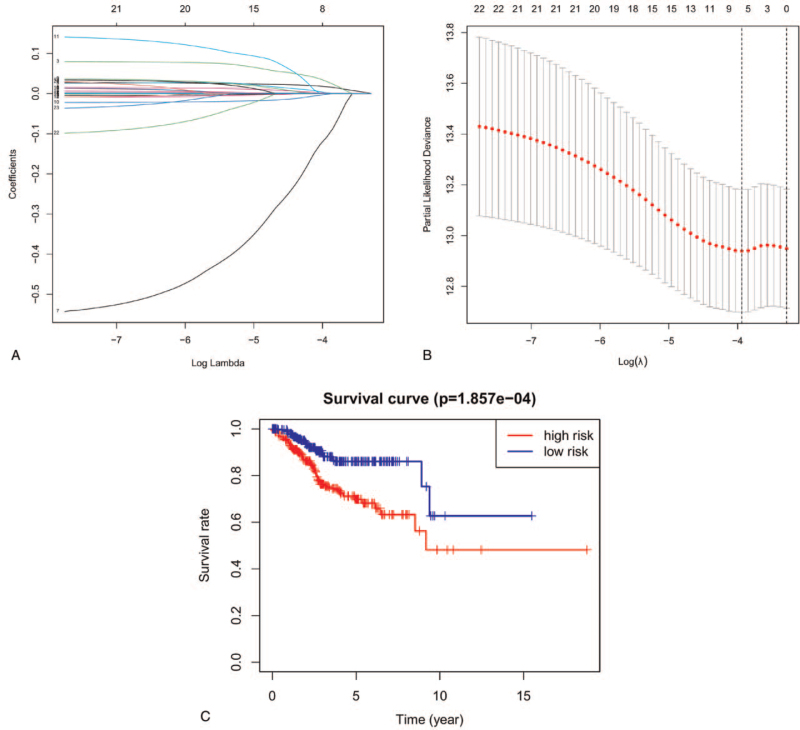
The risk signature of UCEC consisting of m6A regulators. A and B, The coefficients calculated by Lasso Cox regression analysis. C, Kaplan–Meier overall survival curve between high-risk group and low-risk groups with UCEC. UCEC = uterine corpus endometrial carcinoma.

We performed a Lasso Cox regression algorithm to analyze the 23 m6A regulators based on the minimum criteria and penalization parameter lambda (λ) (see Fig. 5A and B). According to the risk scores obtained from LASSO Cox regression analysis, we identified 8 regulators as risk signatures (risk score=0.017LRPPRC+0.001IGFBP2+0.030KIAA1429+0.001IGFBP3+0.002FMR1-0.109METTL14-0.002YTHDF2). To further validate the prognostic ability of our risk signature for UCEC, we stratified the data retrieved from the TCGA dataset into high-risk and low-risk groups according to median of risk score. Next, we plotted the corresponding survival curve (see Fig. 5C). We found that there was a significant difference in the OS curve between the 2 groups, and it was lower in the high-risk group (*P* = 1.857e−04). These results supported the idea that the 8 m6A regulators could act as a prognostic predictor in UCEC.

### 3.5 Validation of prognostic value of the risk signature

**Figure 6:**
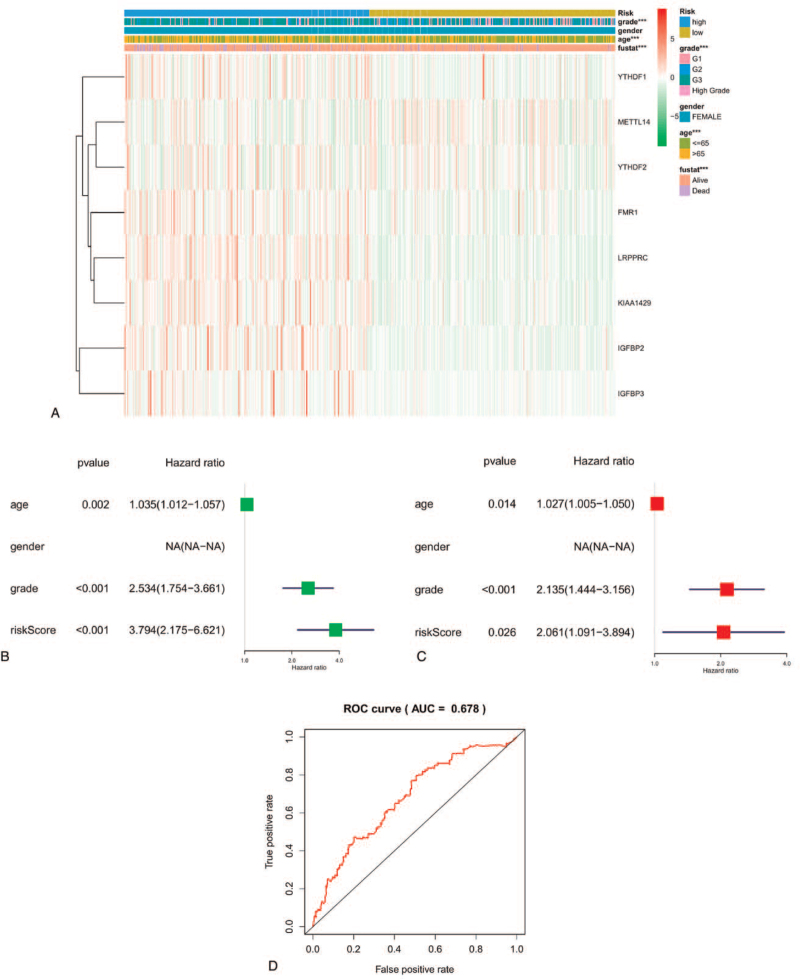
Prognosis value of the risk signature. A, Comparison of expression of 8 m6A regulators and clinical characteristics between high-risk and low-risk groups. B, Univariate Cox regression analysis for patients from TCGA datasets. C, Multivariate Cox regression for overall survival of patients from TCGA datasets. ^∗^*P* < .05, ^∗∗^*P* < .01, and ^∗∗∗^*P* < .001. D, ROC curve showed the predictive value of the risk signature. TCGA = the Cancer Genome Atlas database.

To examine the prognostic value of our risk signature, we compared the expression levels of the identified 8 m6A regulators and several clinicopathologic features, such as age, gender, fustat, and grade between 2 groups. According to the heat map, significant differences were observed between the 2 groups in terms of age (*P* < 0.001), grade (*P* < 0.001), and fustat (*P* < 0.001) (see Fig. 6A). We also found that *YTHDF1, FMR1, LRPPRC, KIAA1429, IGF2BP2,* and *IGF2BP3* were highly expressed in the high-risk group, while *METTL14* and *YTHDF2* were poorly expressed. Univariate cox analysis suggested that age (*P* = 0.002, HR = 1.035, 95% CI = 1.012–1.057), grade (*P* < 0.001, HR = 2.534, 95% CI = 1.753–3.661), and risk score (*P* < 0.001, HR = 3.794, 95% CI = 2.175–6.621) were important influencing factors of OS rate (see Fig. 6B). Moreover, multivariate cox analysis suggested that age (*P* = 0.014, HR = 1.027, 95% CI = 1.005–1.050), grade (*P* < 0.001, HR = 2.135, 95% CI = 1.444–3.156), and risk score (*P* = 0.026, HR = 2.061, 95% CI = 1.091–3.894) could significantly influence OS rate (see Fig. 6C). Furthermore, the AUC was 0.678 (Fig. 6D), which also indicated a good predictive performance.

**Figure 7:**
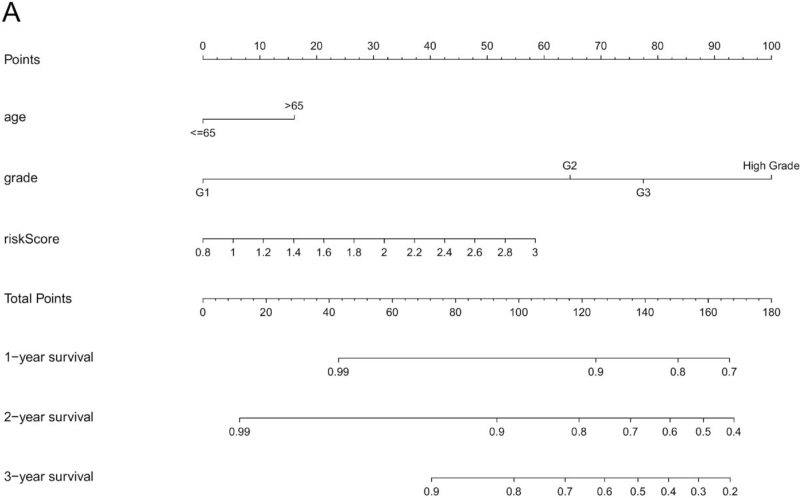
The predictive nomogram of UCEC patients. UCEC = uterine corpus endometrial carcinoma.

To better leverage the risk signature, we constructed a predictive nomogram to calculate the 1-year survival, 2-year survival, and 3-year survival of UCEC patients (see Fig. 7A). Results showed that the nomogram made the signature more intuitive and effective.

## 4 Discussion

M6A methylation has been considered to be the most universal internal cotranscriptional modification in mRNA or long non-coding RNA (lncRNA) in eukaryotes since its discovery in 1974.^[12]^ It mainly interacts with three classes of regulators: the m6A methyltransferases, known as writers; m6A-binding proteins, also called reader; and the demethylases, known as eraser.^[13]^ Plenty of studies have demonstrated the crucial role of m6A RNA methylation regulators in physiological and pathological processes, especially in the occurrence and development of human cancers.^[14–16]^

The rapid development of bioinformatics technology has led to the identification of various prognostic risk signatures consisting of m6A regulators in various types of tumors. Wu et al ^[17]^ used univariate and LASSO Cox regression analysis to develop a m6A regulator prognostic signature composed of *HNRNPC, METTL3, HNRNA2B1, IGF2BP1,* and *IGF2BP2* for lung adenocarcinoma based on RNA-seq, clinicopathological, and single nucleotide variation data retrieved from the TCGA database.^[17]^ Also, Yang et al^[18]^ found that *HNRNPC* and *KIAA1429* can be regarded as potential prognostic markers in papillary renal cell carcinoma by using the similar method. However, only few studies have investigated the prognostic value of m6A regulators in UCEC. Therefore, it is vital to establish the m6A-related prognostic signature for UCEC patients.

To build the risk signature, we chose 23 m6A regulators and compared their expression in normal and tumor samples obtained from TCGA database. Results showed that most of them were differentially expressed in UCEC tissues. Based on the LASSO Cox regression, we identified 8 m6A regulators involving *LRPPRC, IGF2BP2, KIAA1429, IGF2BP3, FMR1, YTHDF1, METTL14,* and *YTHDF2,* which were associated with UCEC progression. The 8 m6A regulators should be considered as the most important result in this study because their expression levels can help us evaluate the prognosis of UCEC patients. We then stratified patients into high-risk and low-risk groups, and combined univariate Cox regression analysis, multivariate Cox regression analysis, and ROC curves to verify the prognostic value of the risk signature. It is worth noting that the low-risk group indeed had a higher 5-year survival rate compared with the high-risk group. Therefore, these observations suggested that this risk signature of eight m6A regulators has potential prognostic value for UCEC patients.

Among these regulators, *LRPPRC, IGF2BP2, I*G*F2BP3, YTHDF1, YTHDF2,* and *FMR1* were all categorized in m6A-binding proteins, which act by conjunction with m6A to read the biological information, mainly for splicing, stabilization, translation, and degradation of RNA.^[19]^ However, some differences still exist between individual regulators. *YTHDF1/2* have synergistic effects on promoting mRNA translation and degradation.^[20]^ Conversely, *IGF2BP2* and *IGF2BP3* try to rescue m6A-modified mRNAs from degradation.^[21]^ Moreover, *LRPPRC* is a multifunctional gene. It has not only been identified as a cofactor for Eukaryotic initiation factor 4E that is central for mRNA translation, but also plays an essential role in mitochondrial energy metabolism by regulating expressions of the mitochondrial DNA-coded mRNAs and peroxisome proliferator-activated receptor coactivator 1-alpha.^[22]^ On the other hand, *FMR1* is usually involved in RNA modification functions such as splicing, nuclear export, and translation in collaboration with *YTHDF2*.^[23]^ It is also regarded as a risk marker of developing Fragile X-associated tremor/ataxia syndrome.^[24]^

Regrettably, there is little data on the role of these genes in UCEC. Shen et al ^[25]^ conducted an in vitro cell experiment which showed that *YTHDF2* promoted cell proliferation and apoptosis in UCEC tissues by degrading m6A-modified lncRNA *FENDRR* and increasing the protein level of SRY-related HMG box transcription factor 4. Although currently there is no direct evidence to clarify the mechanism of action of the other regulators in UCEC, a few studies have reported that they have the ability to impact development of many kinds of tumor. Their mechanism is probably implemented through regulating the stability and transcription of target mRNAs, microRNAs, and lncRNAs. Various target RNAs act via different kinds of signaling pathways to influence cell migration and adhesion, actin cytoskeleton remodeling, and immune microenvironment, ultimately achieving their purpose.^[26–29]^ For example, *METTL14* exhibits pro-apoptosis actions by enhancing autophagy regulated by the mechanistic target of rapamycin pathway in pancreatic cancer.^[30]^ In addition, *KIAA1429* has been shown to affect cell proliferation, migration, invasion, and cell cycle of lung adenocarcinoma by regulating *MUC3A* expression and promoting metastasis of tumor cells by downregulating *ZEB1* in liver cancer.^[31,32]^ Over the years, only 5 m6A regulators, including *FTO, IGF2BP1, YTHDF2, ALKBH5,* and *WTAP* have been shown to play defined roles in UCEC.^[9,25,33–35]^ Apparently, the association between m6A regulators and UCEC is still poorly understood and worthy of further exploration.

This study successfully identified m6A-related prognostic markers of UCEC and explored the functions of related genes, thereby providing a new direction for the study of UCEC pathogenesis from the modification mechanism of m6A. However, this study had some limitations. Firstly, the AUC value of the risk signature was 0.6to 0.7, which means the sensitivity and specificity of this signature could be increased in the future. Secondly, stratified analysis according to cancer type could not be performed because of the small sample size. Thirdly, our results were based on bioinformatics analysis of published data. Therefore, many repeated experiments or large-scale, prospective, and multicenter studies are required to validate the results. In the future, our studies will focus on experimental verification of the conclusion of this study and elucidation of the specific mechanism of m6A regulators in UCEC.

## 5 Conclusion

In summary, we systematically evaluated the expression of 23 m6A regulators in UCEC samples, and build a 8-m6A regulators risk signature and predictive nomogram for patients with UCEC. Furthermore, the regulators used to establish the risk signature may be potential targets for UCEC treatment and prevention.

## Acknowledgments

The authors thank Home for Researchers editorial team (www.home-for researchers.com) for English language editing and Mr Weijun Zheng for providing statistical methodology consultation.

## Author contributions

**Conceptualization:** Qingge Guo.

**Data curation:** Ying Zhao, Qingge Guo.

**Methodology:** Chenyun Miao, Xiaojie Fang.

**Software:** Xiaojie Fang, Ying Zhao.

**Supervision:** Qingge Guo.

**Validation:** Yun Chen.

**Writing – original draft:** Chenyun Miao, Yun Chen, Qingge Guo.
